# Graphene Oxide/Styrene-Butadiene Latex Hybrid Aerogel with Improved Mechanical Properties by PEI Grafted GO and CNT

**DOI:** 10.3390/gels9050419

**Published:** 2023-05-16

**Authors:** Zetian Zhao, Lina Zhang, Yinghu Song, Lichun Ma, Jialiang Li, Min Zhao, Xueliang Ji, Jianfei Gao, Guojun Song, Xiaoru Li

**Affiliations:** Institute of Polymer Materials, School of Material Science and Engineering, Qingdao University, No. 308 Ningxia Road, Qingdao 266071, China

**Keywords:** synergistic effect, mechanical properties, graphene oxide aerogel, polyethyleneimide, styrene-butadiene latex

## Abstract

Graphene oxide aerogel (GOA) has wide application prospects due to its low density and high porosity. However, the poor mechanical properties and unstable structure of GOA have limited its practical applications. In this study, polyethyleneimide (PEI) was used to graft onto the surface of GO and carbon nanotubes (CNTs) to improve compatibility with polymers. Composite GOA was prepared by adding styrene-butadiene latex (SBL) to the modified GO and CNTs. The synergistic effect of PEI and SBL, resulted in an aerogel with excellent mechanical properties, compressive resistance, and structural stability. When the ratio of SBL to GO and GO to CNTs was 2:1 and 7:3, respectively, the obtained aerogel performance was the best, and the maximum compressive stress was 784.35% higher than that of GOA. The graft of PEI on the surface of GO and CNT could improve the mechanical properties of the aerogel, with greater improvements observed with grafting onto the surface of GO. Compared with GO/CNT/SBL aerogel without PEI grafting, the maximum stress of GO/CNT–PEI/SBL aerogel increased by 5.57%, that of GO–PEI/CNT/SBL aerogel increased by 20.25%, and that of GO–PEI/CNT–PEI/SBL aerogel increased by 28.99%. This work not only provided a possibility for the practical application of aerogel, but also steered the research of GOA in a new direction.

## 1. Introduction

Graphene is a typical two-dimensional (2D) material with a single atomic layer thickness consisting of *sp*^2^ hybrid structures, which is one of the thinnest 2D materials—the thickness is about 0.35 nm—in the world [1,2]. Graphene has become a research hotspot due to its excellent properties such as ultra-high elastic modulus, excellent conductivity, preferable thermal conductivity and large specific surface area, which can be applied in sensors [3], electromagnetic shielding [4,5], supercapacitors [6,7] and other fields. Graphene oxide (GO) is an important derivative of graphene. The structure of GO is similar to graphene, and GO has rich oxygen-containing functional groups, which can be used as the binding sites of nanoparticles. The appearance of a folded structure in GO could effectively prevent the agglomeration of other nanoparticles. The presence of oxygen-containing functional groups in GO makes the carbon layer negatively charged and cations can easily enter between the GO sheets, widening the spacing between the GO layers. Therefore, GO can be used as an ideal raw material in the preparation of composite materials [8,9,10]. Additionally, these composite materials have been widely used in aerospace, construction, energy storage, catalysis, sensors and many other fields [11,12,13]. However, graphene sheets tend to accumulate and agglomerate due to the strong π–π interaction between graphene sheets and Van der Waals forces. The effective area of graphene has been greatly reduced, limiting its application in some fields. At present, an ideal solution is to combine 2D graphene oxide with 1D carbon nanotubes (CNTs) to prepare graphene oxide aerogel (GOA) with a 3D structure. The axial dimensions of CNTs are micrometers, while the radial dimensions can be up to nanometers. CNTs have extremely high mechanical strength and elasticity due to their large aspect ratio [14,15]. Therefore, this not only solves the problem of poor dispersion of GO, but also improves the specific surface area of the material, thus effectively improving the mechanical properties of the material.

Three-dimensional graphene oxide aerogel (GOA) consists of 2D GO sheets as the base unit, which are stacked on top of each other to form porous materials with well-developed internal voids [16]. GOA not only has the excellent properties of aerogel with low density and well-developed porosity, but also has the excellent mechanical and electrical properties of GO, so it has wide application prospects in adsorption, sensors, energy storage, catalyst carriers and other fields [17,18,19,20,21,22]. However, the obtained aerogel by directly self-assembly of GO and CNTs has weak mechanical properties and is prone to collapse when subjected to external stresses. Therefore, it is necessary to improve the specific surface area and porosity of GOA. In addition, in order to improve the mechanical properties of GOA, the gap between GO and CNTs should be improved. It is currently still a challenge to increase the effective specific surface area and enhance the mechanical properties.

There has been a great deal of effort to research enhanced GOA. On the one hand, small organic molecules can be grafted onto the surface of GO and CNTs to improve the interface compatibility between the phases, to improve the structural stability and mechanical properties of GOA. Nishanth et al. [23] introduced nitrogen-containing groups on the GO surface by grafting boron nitride on the surface, which reduced the surface energy and surface tension of GO to improve the dispersion and the compressive strength of GO aerogel. The compressive strength of the aerogel prepared by GO–BN was 61.5% higher than that of the original aerogel. Liu et al. [24] modified graphene with methyl triethoxyl-silane to prepare an enhanced aerogel with excellent compression resistance and up to 1000 compression cycles. Donchak et al. [25] grafted MPI on the surface of NH_2_-CNT, and prepared CNT with polybutylene terephthalate and polytetramethylene oxide into composite nanomaterials. The nanocomposites had good mechanical properties and thermal properties, and their maximum breaking tension was up to 32.9 MPa. Polyethyleneimine (PEI) is a water-soluble polymer with many amino groups, which can be combined with materials with active groups [26,27]. By grafting PEI onto the surface of GO and CNTs, abundant surface-active functional groups can be introduced to improve the surface roughness of the sample and improve the dispersion of GO and CNTs [28]. On the other hand, many researchers have prepared composite aerogels by adding polymers or organic compounds, which greatly improved the compression properties and enhanced the mechanical properties. Ren et al. [29] prepared polyimide/graphene aerogel, which increased the maximum stress while providing oil and water adsorption properties. However, when the compression was too large, the structure of the aerogel was destroyed and the aerogel would still collapse. Therefore, more effective fillers should be used to enhance the mechanical properties of aerogel. The latex particles have excellent elasticity and good dispersion in GO solution. High strength aerogel with high orientation pore structure, high flexibility and high elasticity was prepared by using latex as filler. Zhang et al. [30] prepared GO/NBL composite aerogel with a light weight and high toughness by blending and heat-treating GO with natural latex using SDS as surfactant. Through the introduction of natural latex particles, a relatively strong 3D structure was successfully constructed, which not only has excellent impact resistance and tensile resistance, but also has strong adsorption of water. When the strain is 50%, the maximum stress of GA/NRL can reach 6 KPa. The practical application of GOA has been solved successfully.

A composite aerogel with high compressibility and stable structure has been designed in this paper. The modified GO–PEI and CNT–PEI were obtained by grafting PEI onto the surface of GO and CNT, respectively. Styrene-butadiene latex was used as reinforcement. PEI can significantly improve the dispersion of GO and CNT, and SBL has better mechanical properties. Chemical modification of GO and CNT with PEI was performed to enhance the interfacial compatibility between GO/CNT and polymers. The addition of SBL increased the cross-linking points of the aerogel. The synergistic effect of both methods improved the mechanical properties of graphene oxide aerogel. The enhanced aerogels prepared by this method will have a wide range of applications, such as insulation materials, adsorbent materials, electromagnetic shielding materials, and energy storage materials. This study provides a new approach for the preparation of functional composite aerogels.

## 2. Results and Discussion

### 2.1. Surface Chemical Structure Analysis of GO and CNTs’ Materials

**Figure 1:**
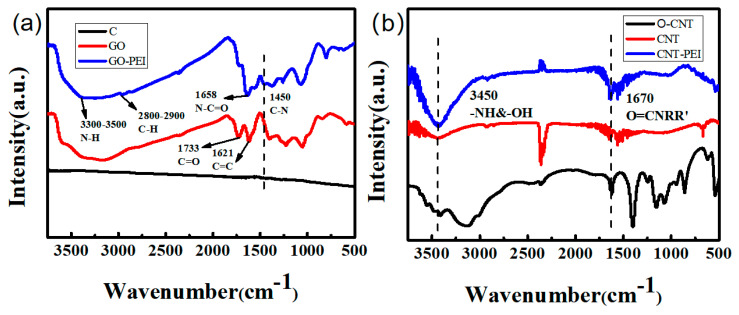
FT-IR spectra of various samples: (**a**) C/GO/GO–PEI, (**b**) O–CNT/CNT/CNT–PEI.

The composition of the samples was determined by Fourier transform infrared absorption spectrometry (FT-IR). As shown in Figure 1a, for GO–PEI, the new peak at 2900–2800 cm^−1^ is the stretching vibration peak of C-H in PEI, and the stretching vibration peak of N-H is around 3300–3500 cm^−1^. The absorption peak of the amide bond formed by the carboxyl group in GO and the amino group of PEI is 1658 cm^−1^, C=O and N-C=O were obscured by the formation of N-C=O. Meanwhile, the absorption peak of C-N bond appears at 1450 cm^−1^. Figure 1b shows the FT-IR spectra of original CNT (O-CNT), oxide CNT and CNT–PEI. Obviously, the peak of CNT–PEI at 3450 cm^−1^ is stronger than of CNT, which is attributed to tensile vibration of O-H and N-H. The peak of CNT–PEI at 1670 cm^−1^ was caused by the stretching vibration of the amide at O=CNRR’. The appearance of the new characteristic peaks and the enhancement of the original characteristic peaks successfully demonstrated the grafting of PEI on the surface of GO and CNT.

**Figure 2:**
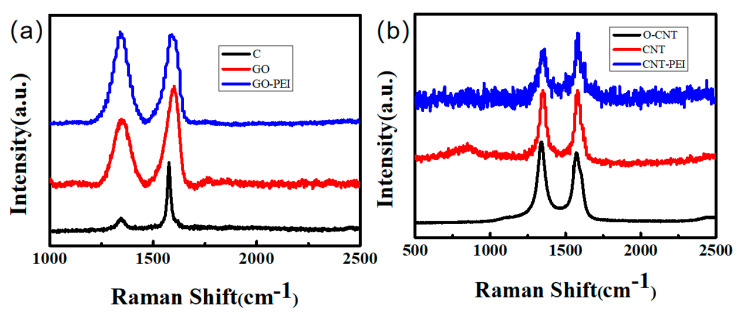
Raman spectra of various samples: (**a**) C/GO/GO–PEI, (**b**) O–CNT/CNT/CNT–PEI.

Figure 2a shows the Raman spectra of C, GO, and GO–PEI. The relative ratio *R*(I_D_/I_G_) is used to judge the degree of order of the carbon structure. A higher *R*-value indicates a lower degree of order and a lower degree of graphitization. The *R*-value of GO (0.885) is much higher than that of graphite powder (0.26), indicating that the degree of disorder of GO is much higher than that of graphite powder. This is because the surface of GO contains a large number of hydroxyl and carboxyl groups, which greatly increases the layer spacing and degree of disorder. The *R*-value of GO–PEI changed from 0.885 to 1.218, indicating that the grafting of PEI resulted in the appearance of new functional groups on the GO surface, which further increased the layer spacing between GO layers. Figure 2b shows the Raman spectra of O–CNT, CNT and CNT–PEI. The *R*-values of O–CNT, CNT and CNT–PEI were 1.076, 1.077 and 1.131, respectively. The grafting of PEI improved the disorder degree of CNT and thus increased the *R*-value of CNT–PEI, but the increase range was limited. This might be because surface grafting has no significant effect on the internal structure of CNT. The increase in *R-*value indicated that a new chemical bond appeared on the surface of the sample, which destroyed the original regularity, and proved that the graft of PEI was successful.

**Figure 3:**
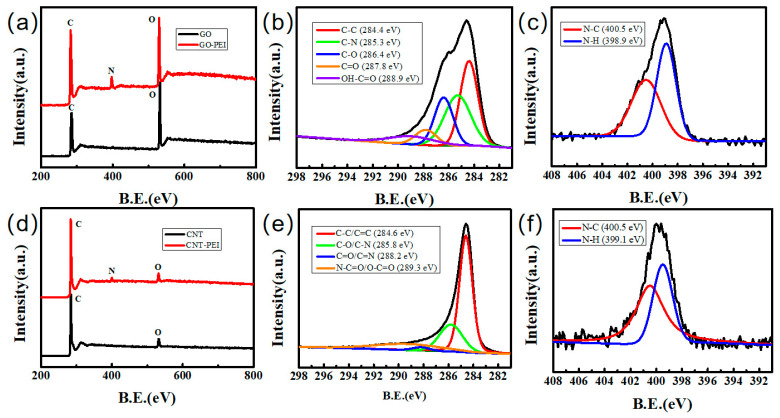
XPS survey spectra of various samples: (**a**) wide scan of GO–PEI, (**b**) C 1s of GO–PEI, (**c**) N 1s of GO–PEI, (**d**) wide scan of CNT–PEI, (**e**) C 1s of CNT–PEI, (**f**) N 1s of CNT–PEI.

X-ray photoelectron spectroscopy (XPS) measurement was conducted to determine the surface chemical composition. As shown in Figure 3a,d, GO had more oxygen-containing groups than CNT, so the XPS spectra of GO displayed a higher O peak. The presence of C, N and O elements in GO–PEI is confirmed by the XPS spectrum in Figure 3a. A new N 1s peak appeared at 400 eV. Five binding peaks of C 1s of GO–PEI, namely C–C, C–N, C–O, C=O and O–C=O appeared at 284.4 eV, 285.3 eV, 286.4 eV, 287.8 eV and 288.9 eV, respectively. N 1s can be divided into two peaks: N–C and N–H. Figure 3d also shows the emergence of new N peaks. As shown in Figure 3e, the positions of CNT–PEI characteristic peaks C–C/C=C, C–O/C-N, C=O/C=N and N–C=O and O–C=O were 284.6 eV, 285.8 eV, 288.2 eV and 289.3 eV, respectively [30]. The introduction of PEI increased the richness of functional groups on the sample surface. Therefore, the dispersion of samples was significantly improved.

### 2.2. Morphology Analysis

**Figure 4:**
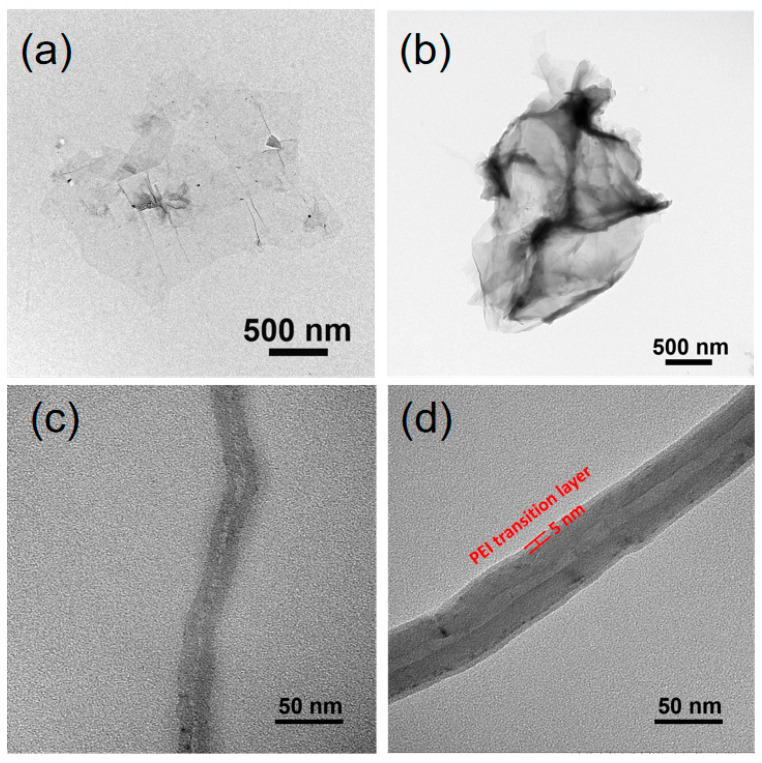
TEM images of (**a**) GO, (**b**) GO–PEI, (**c**) CNT, (**d**) CNT–PEI.

The TEM images of GO, GO–PEI, CNT and CNT–PEI are shown in Figure 4. Compared with GO, the GO–PEI sheet is thicker (Figure 4b). It can be seen that there was an obvious transition layer of 5 nm thickness on the surface of CNT, which made CNT thicker. The transition layer was mainly composed of PEI. These phenomena indicated that PEI was successfully grafted to the surface of GO and CNT.

**Figure 5:**
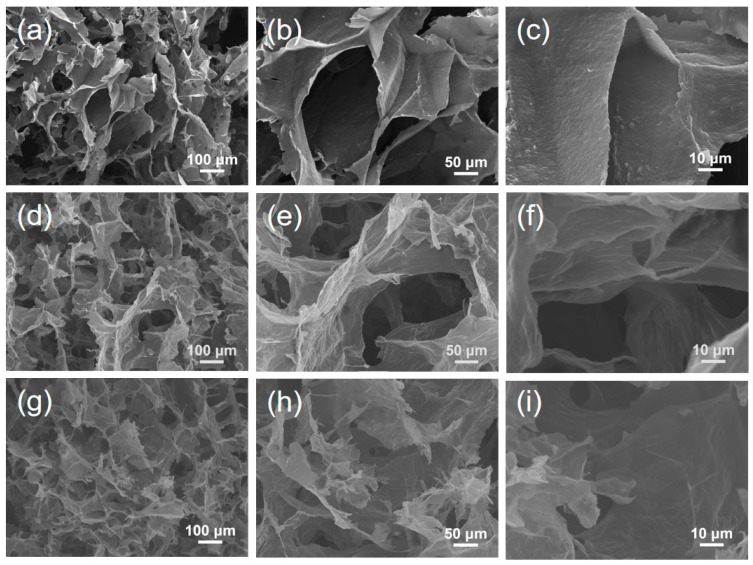
SEM images of aerogels: (**a**–**c**) GO/CNT/SBL (SBL:GO = 2:1), (**d**–**f**) GO/CNT–PEI/SBL (SBL:GO = 2:1), (**g**–**i**) GO–PEI/CNT–PEI/SBL (SBL:GO = 2:1).

The SEM images of GO/CNT/SBL, GO/CNT–PEI/SBL GO–PEI/CNT–PEI/SBL aerogel are shown in Figure 5, the scanning signal used was secondary electron. As shown in the Figures, the aerogel of non-grafted PEI presented a 3D honeycomb structure, while the aerogel of grafted PEI presented a checkerboard structure, and the 3D structure of grafted PEI was more regular. By comparison, it can be seen that the morphology of GO/CNT–PEI/SBL aerogel was not different from that of GO–PEI/CNT–PEI/SBL aerogel, which proved that the grafting of PEI on different materials did not affect the morphology of the aerogel. According to the BET curves, it could be found that PEI grafting had no significant influence on the surface area of GO and the pore size of the aerogel (Figure S4). The regular 3D structure could provide better mechanical properties in the aerogel.

### 2.3. Mechanical Properties

**Figure 6:**
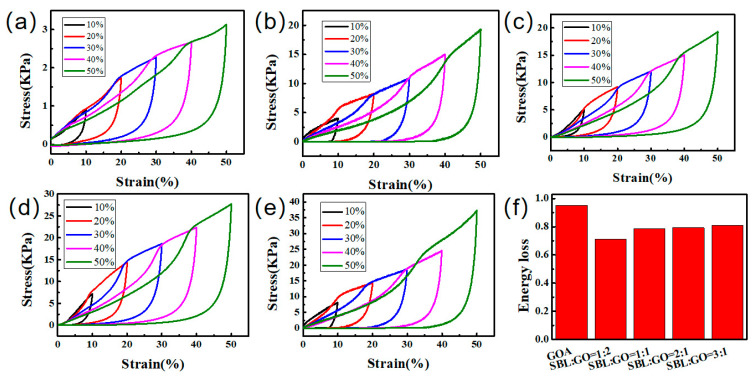
Compression curves of (**a**) GOA, (**b**) SBL:GO = 1:2, (**c**) SBL:GO = 1:1, (**d**) SBL:GO = 2:1, (**e**) SBL:GO = 3:1, (**f**) EL curves at 50% compression.

The effects of pre-vulcanization and accelerator on aerogel properties were discussed, and it was determined that the subsequent vulcanization process would significantly improve the properties of aerogel if the accelerator was added to the prevulcanized SBL (the mechanical properties of the pre-vulcanization, vulcanization and accelerator samples were included in the supplementary material, refer to Figure S2). The influence of SBL to GO mass ratio on aerogel performance was then discussed. Figure 6 shows the compression curves of GO/CNT/SBL aerogel prepared with different ratios of GO and SBL, and the data summarized from Figure 6 is shown in Table 1. Compared with GOA, the mechanical properties were enhanced with the increase in latex content (Figure 6a–e), and the higher the latex content, the more energy was lost. SBL:GO = 2:1 and SBL:GO = 3:1 aerogels had better mechanical properties than other aerogels. Compared with GOA, the maximum stress was increased by 784.35% and 1089.80%, respectively. In addition, the energy loss of SBL:GO = 3:1 aerogel was large, resulting in local collapse of the aerogel. In contrast, the aerogel with SBL:GO = 2:1 did not show obvious local collapse after compression (Figure S1). This indicated that the filling of latex effectively reduced the energy loss of GOA. However, the energy loss of aerogel increases with increasing latex content. Therefore, the latex content in aerogel should be controlled in an appropriate range. In summary, the aerogel prepared at the corresponding ratio of 2:1 not only has good mechanical properties but also does not easily collapse after compression, with good structural stability and low energy loss, which is a good ratio requirement.

**Figure 7:**
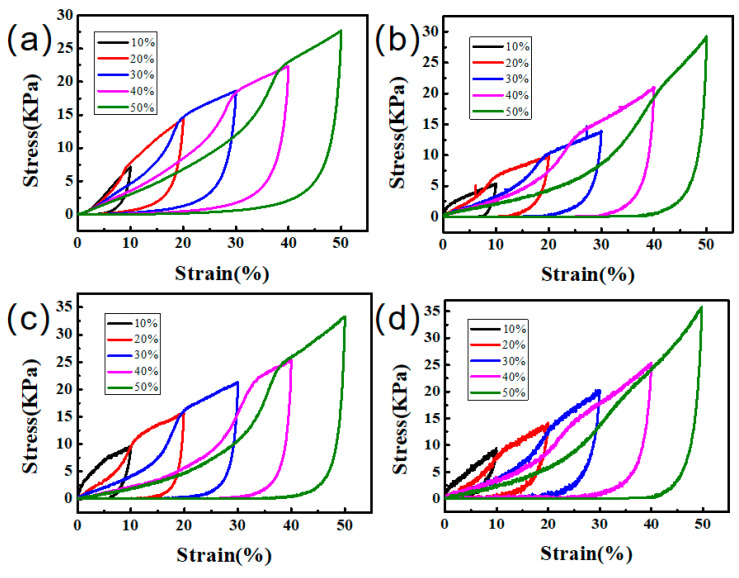
Compression curves of (**a**) GO/CNT/SBL, (**b**) GO/CNT–PEI/SBL, (**c**) GO–PEI/CNT/SBL, and (**d**) GO–PEI/CNT–PEI/SBL.

The mechanical properties of different aerogel samples were examined by compression tests as shown in Figure 7 and Table 2. The mass ratio of SBL to GO was 2:1. By comparing Figure 7a,b, it could be found that after grafting PEI to the surface of CNT, the mechanical properties of aerogel were slightly improved (by 5.57%). Compared to the non-grafted aerogel, it was found that the compression strength of GO–PEI/CNT/SBL aerogel was significantly improved by 20.25% (Figure 7c). As shown in Figure 7d, when PEI was grafted with GO and CNT at the same time, the maximum stress of aerogel increased by 28.99%. This is mainly because GO, as the skeleton of the aerogel, has more contact with latex and plays a major role in the mechanical properties of the aerogel, while CNT only plays the role of lap bonding and has no significant change to the mechanical properties of aerogel.

### 2.4. Thermal Properties

**Figure 8:**
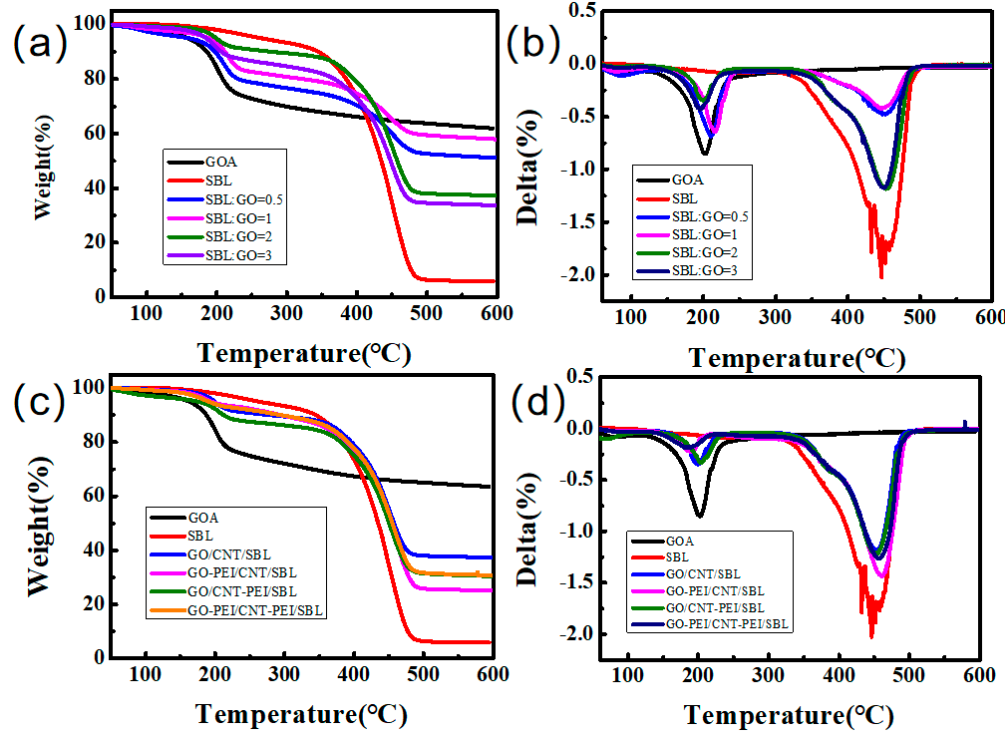
Thermal analysis: (**a**) TGA and (**b**) DTG curves of samples of various mass ratios, (**c**) TGA and (**d**) DTG curves of samples grafted with PEI.

Figure 8 shows the TGA curve and DTG curve of the samples, and Table 3 shows the residual mass ratio of samples after thermal decomposition. The weight loss at around 200 °C is mainly due to the removal of the remaining water molecules in the GO and the decomposition of a few unstable functional groups, while the weight loss at around 450 °C is mainly due to the decomposition of SBL in the aerogel. Therefore, GOA has only one weight loss at 200 °C and SBL has only one weight loss at 450 °C. At 450 °C, the weight loss of SBL:GO = 1:1 sample is 14.12%, while the weight loss of SBL:GO = 2:1 and SBL:GO = 3:1 samples are 36.83% and 43.07%, respectively. As shown in Figure 8b,d, the weight loss temperature of PEI grafted GO/CNT/SBL aerogel did not change significantly, mainly because the chemical composition of aerogel did not significantly change after PEI grafting. The TG of PEI grafted on CNT samples was 43.02%, and that of PEI grafted on GO samples was 50%, which was caused by the decomposition of nitrogen-containing groups in PEI. However, the final residue quality of the aerogel grafted by PEI to GO and CNT is improved, with a mass loss of only 47.30%, possibly due to the formation of more stable nitrogen-containing bonds during self-assembly. It can be seen from Figure 8c,d that the higher the nitrogen content in the sample, the slower the decomposition of carbon-containing groups at 200 °C and the faster the decomposition of nitrogen-containing groups at 450 °C. According to Figure 8c, it could be calculated that the graft rate of GO–PEI was 12.50%, and the graft rate of CNT–PEI was 6.87%. In addition, changing the ratio of GO and SBL had little effect on the glass transition temperature of the composite erogel, while grafting PEI increased the glass transition temperature of the composite aerogel (Figure S3). This is because the glass transition temperature of aerogel is mainly provided by the styrene-butadiene latex, the addition of GO and CNT will not affect the structure of the styrene-butadiene latex, but PEI has a larger molecular weight and crystallization phenomenon will appear in the heating process, thus improving the glass transition temperature of the aerogel. The introduction of nitrogen-containing groups can indeed improve the mechanical properties of aerogel, but its effect on thermal properties should also be considered.

## 3. Conclusions

In conclusion, we successfully prepared a GO enhanced aerogel with excellent mechanical properties, thermal properties and compression resistance through the synergistic strengthening effect of PEI and SBL. GO acted as the backbone, while SBL enhanced the cross-linking points of the GO sheets. The maximum stress of GO/CNT/SBL aerogel was 784.35% higher than that of the GO/CNT aerogel. Furthermore, the maximum stress of GO–PEI/CNT–PEI/SBL aerogel was increased by 28.99% compared to GO/CNT/SBL aerogel, indicating that PEI grafting improved the compatibility of GO, CNT and SBL, further improved the mechanical properties of aerogel, and realized the synergistic enhancement effect of PEI grafting and SBL filling. The prepared aerogel can be mixed with other polymers to prepare graphene composites and used as the carrier for other nanoparticles, which adds additional functions to the aerogel, expands the application possibilities of the aerogel, and provides more research ideas for the practical application of graphene aerogel.

## 4. Materials and Methods

### 4.1. Materials

Graphite: particle size of 0.5~1 μm, carbon content of 90~99.99%, Qingdao Haida graphite factory (Qingdao, China). Carboxylated carbon nanotubes (CNT): Institute of polymer materials, Qingdao University, diameter 10~20 nm, length > 5 μm, purity > 97%, Shenzhen Nanotube Company (Shenzhen, China). Polyethyleneimine polymer (PEI): The molecular weight of 600, Shanghai Aladdin Biochemistry Technology Co., Ltd. (Shanghai, China). Styrene-butadiene latex (SBL): TSC is 221 mg/mL, Foshan Jinjia New Material Technology Co., Ltd. (Foshan, China). N,N′-Dicyclohexylcarbodiimide (DCC): Shanghai Aladdin Biochemistry Technology Co., Ltd. (Shanghai, China). N-Methyl-2-pyrrolidone (DMF), 2,2′-Dithiobis (benzothiazole) (Accelerator DN), N-hydroxysuccinimide (NHS), N-Cydohexyl-2-benzothiazolylsulfenamide (Accelerator CZ), 1-ethyl -(3-dimethylaminopropyl) carbanyl diimide hydrochloride (EDC): Shanghai Macklin Biochemical Technology Co. Ltd. (Shanghai, China).

### 4.2. Methods

#### 4.2.1. Preparation of Graphene Oxide

The modified Hummers method was used in this experiment [31]. First, graphite, NaNO_3_ and concentrated sulfuric acid were added to a three-mouthed bottle. After 30 min pre-oxidation by mechanical stirring in an ice water bath, KMnO_4_ was added for a 2 h reaction, and the temperature was raised to 35 °C for a 17 h reaction. After the reaction, 10:1 H_2_O and H_2_O_2_ were added and mechanically stirred for 20 min. The suspension was stripped by simple ultrasound for 30 min and the supernatant was removed. The mixture was centrifuged and cleaned with 20% HCl solution until the supernatant was added to 0.1 mol/L BaCl_2_ solution without precipitation. It was then washed with deionized water until nearly neutral, to prepare a GO suspension.

#### 4.2.2. Preparation of GO–PEI

A 1 mg mL^−1^ GO suspension was prepared by dissolving 300 mg of GO into 500 mL of distilled water in a round-bottomed flask. The reaction flask was placed in an ultrasonic water bath for 30 min to make the suspension evenly dispersed. The 5:6 mass ratio of NHS and EDC was added to the GO suspension and stirred at room temperature to dissolve. Then, 60 mL 10 g L^−1^ polyethyleneimine aqueous solution was added to the reaction bottle dropwise. After addition, the simple ultrasonic reaction lasted for 1h, and the reaction was left overnight at room temperature. Finally, the reaction mixture was centrifuged, washed with anhydrous ethanol five times, and freeze-dried to obtain the GO–PEI samples.

#### 4.2.3. Preparation of CNT–PEI

A total of 4 mL of PEI was dissolved in 40 mL DMF, and 0.5 g CNTs were added to the above solution after 30 min of simple ultrasound. The above mixture was kept in an N_2_ atmosphere at room temperature for 36 h. After that, PEI and DMF were added to the reaction solution in the same proportion for ultrasonic treatment. Then 0.85 g DCC and three molecular sieves were added successively, and the reflux reaction was heated in the oil bath for 24 h. After the reaction, CNTs were repeatedly cleaned with distilled water and ethanol and freeze-dried to prepare CNT–PEI.

#### 4.2.4. Preparation of GO/CNT/SBL Aerogel

**Figure 9:**
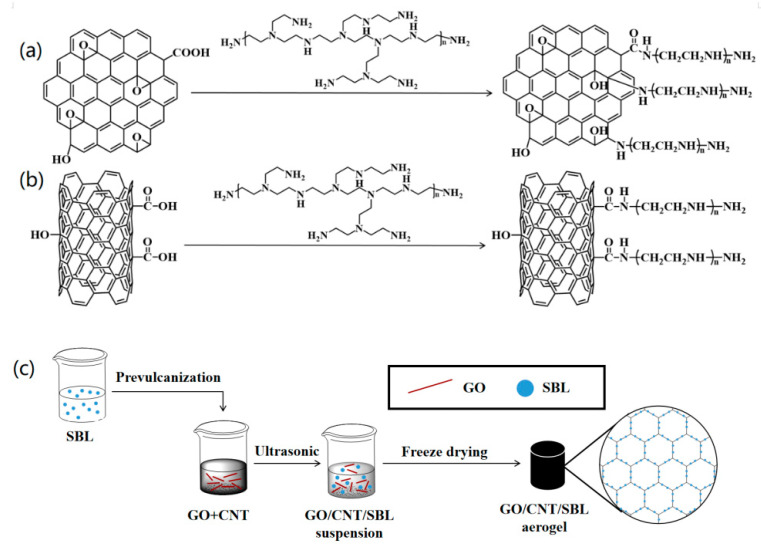
Schematic of preparation for (**a**) GO–PEI, (**b**) CNT–PEI, (**c**) GO/CNT/SBL aerogel.

The vulcanizing agent sulfur (S), accelerator CZ, accelerator DN and ZnO were added to styrene-butadiene latex (SBL) at a mass ratio of 2:2:2:2:5, and pre-vulcanized at 40 °C. The mass ratio of the total mass of the vulcanizing agent mixture to the solid content of SBL was 1:100. The mixture was heated to 65 °C during agitation and placed at room temperature for 24 h to complete the pre-vulcanization. Previous studies had shown that the microstructure and mechanical properties of GO aerogel were the best when the mass ratio of GO to CNT was 7:3 [32]. GO and CNT were dispersed in distilled water after simple ultrasonic treatment. Then precure styrene-butadiene latex was added to GO dispersion solution for ultrasonic treatment. Next, the mixture was freeze-dried for 48 h. After that, GO/CNTs/SBL aerogel was obtained by hot curing at 100 °C for 6 h. The preparation method and process of other aerogels such as GO–PEI/CNT/SBL, GO/CNT–PEI/SBL and GO–PEI/CNT–PEI/SBL were the same as the above preparation method. The preparation of GO–PEI, CNT–PEI and their aerogels are shown in Figure 9. The formula of each sample is shown in Table 4.

### 4.3. Characterization

Fourier transform infrared absorption spectroscopy (FTIR) was used to scan the samples under an infrared spectrometer probe with a resolution of 8 cm^−1^ and a test range of 500–4000 cm^−1^ (NEXUS-670, Nicole Company). Raman spectrum test conditions were: 10% power, frequency range 1000–3000 cm^−1^ and 1000–2000 cm^−1^, respectively. X-ray photoelectron spectroscopy (XPS) patterns were used to analyze the chemical composition and relative element content of samples to determine the composition of the functional groups on the surface of samples (220I-XL, ESCA Lab). The micromorphology of GO/CNT/SBL aerogel sections was observed by a JSM-7800F scanning electron microscope produced by JEOL Company in Japan. The compression speed was maintained at 2 mm min^−1^, and the sample height was compressed to 10%, 20%, 30%, 40% and 50% of the original height (WDW-5T Microcomputer Controlled Electronic Testing Machine, Jinan Hengruijin Testing Machine Co., Ltd.). The thermogravimetric test temperature was increased from 50 °C to 600 °C at a rate of 10 °C min^−1^ and was carried out in a nitrogen environment (SDT 650, A Discovery Company).

## Figures and Tables

**Table 1:** Mechanical properties of GO/CNT/SBL aerogel.

Sample Name	10% Compression (KPa)	20% Compression (KPa)	30% Compression (KPa)	40% Compression (KPa)	50% Compression (KPa)
GO/CNT	0.90 ± 0.08	1.73 ± 0.12	2.28 ± 0.11	2.66 ± 0.14	3.13 ± 0.13
GO/CNT/SBL (SBL:GO = 1:2)	4.03 ± 0.24	8.30 ± 0.31	10.92 ± 0.19	15.04 ± 0.26	19.31 ± 0.23
GO/CNT/SBL (SBL:GO = 1:1)	5.23 ± 0.33	9.24 ± 0.41	12.07 ± 0.39	15.03 ± 0.42	19.31 ± 0.35
GO/CNT/SBL (SBL:GO = 2:1)	7.17 ± 0.32	14.37 ± 0.37	18.62 ± 0.42	22.36 ± 0.36	27.72 ± 0.44
GO/CNT/SBL (SBL:GO = 3:1)	8.14 ± 0.29	14.41 ± 0.31	18.79 ± 0.46	24.64 ± 0.32	37.26 ± 0.56

Note: Each sample was tested with three replicates.

**Table 2:** Mechanical properties of GO/CNT/SBL aerogel grafted with PEI.

Sample Name	10% Compression (KPa)	20% Compression (KPa)	30% Compression (KPa)	40% Compression (KPa)	50% Compression (KPa)
GO/CNT/SBL	7.17 ± 0.32	14.37 ± 0.37	18.62 ± 0.42	22.36 ± 0.36	27.72 ± 0.44
GO/CNT–PEI/SBL	5.40 ± 0.21	9.96 ± 0.34	14.66 ± 0.15	21.00 ± 0.27	29.26 ± 0.31
GO–PEI/CNT/SBL	9.54 ± 0.15	15.69 ± 0.27	21.38 ± 0.13	25.37 ± 0.24	33.33 ± 0.23
GO–PEI/CNT–PEI/SBL	9.45 ± 0.22	14.18 ± 0.36	20.24 ± 0.42	25.40 ± 0.33	35.75 ± 0.47

Note: Each sample was tested using three replicates.

**Table 3:** Residual mass ratio of sample after thermal decomposition.

Sample Name	200 °C (%)	450 °C (%)
GOA	75.54	65.70
SBL	98.40	7.05
GO/CNT/SBL (SBL:GO = 0.5)	80.53	53.05
GO/CNT/SBL (SBL:GO = 1)	83.24	60.24
GO/CNT/SBL (SBL:GO = 2)	91.67	38.42
GO/CNT/SBL (SBL:GO = 3)	88.42	34.88
GO–PEI/CNT/SBL	94.39	25.92
GO/CNT–PEI/SBL	88.13	31.55
GO–PEI/CNT–PEI/SBL	93.62	31.69

**Table 4:** Formula of each sample.

Sample Name	SBL:GO
GO/CNT/SBL	1:2
GO/CNT/SBL	1:1
GO/CNT/SBL	2:1
GO/CNT/SBL	3:1
GO–PEI/CNT/SBL	2:1
GO/CNT–PEI/SBL	2:1
GO–PEI/CNT–PEI/SBL	2:1

Note: Mass ratio of GO:CNT in all samples was 7:3.

## Data Availability

Not applicable.

## Supplementary Materials

The following supporting information can be downloaded at: https://www.mdpi.com/article/10.3390/gels9050419/s1, Figure S1: compression and recovery process of GO/CNT/SBL aerogel. Figure S2: compression curves of GO/CNT/SBL aerogel (a) prevulcanization and vulcanization, (b) no prevulcanization or vulcanization, (c) no accelerant added but prevulcanization and vulcanization, (d) no prevulcanization, but vulcanization. Figure S3: DSC curves of various samples: SBL, GO/CNT/SBL aerogel, GO–PEI/CNT–PEI/SBL aerogel. Figure S4: BET curves of various samples: (a) GO/CNT/SBL aerogel, (b) GO-PEI/CNT-PEI/SBL aerogel.
