# Supplementary material for: Graphene Oxide/Styrene-Butadiene Latex Hybrid Aerogel with Improved Mechanical Properties by PEI Grafted GO and CNT
Source: Gels. 2023 May 16;9(5):419. doi: 10.3390/gels9050419 (PMC10217333; doi:10.3390/gels9050419)
Supplement: Supplementary file 1 [file gels-09-00419-s001.zip › gels-2347548-supplementary.pdf]

Supplementary material

Graphene Oxide/Styrene-Butadiene Latex Hybrid Aerogel with Improved Mechanical Properties by PEI Grafted GO and CNT

Zetian Zhao, Lina Zhang, Yinghu Song, Lichun Ma, Jialiang Li, Min Zhao, Xueliang Ji, Jianfei Gao, Guojun Song, Xiaoru Li*

Institute of Polymer Materials, School of Material Science and Engineering, Qingdao University, No. 308 Ningxia Road, Qingdao 266071, China

* Correspondence: lixiaoruqdu@126.com

Figure S1 shows the recovery of the GO/CNT/SBL aerogel after compression. Fig. In Figure S1a-c, the aerogel was compressed to 50% of its original height (from 25 mm to 12.5 mm), and it can be seen that the aerogel returned to its original height after the pressure is removed. In Figure S1d-f, 500g weights were placed on the GO/CNT/SBL aerogel, so that the height of the aerogel changed from 25mm to 16mm, and the aerogel recovered its original height after removing the weights. So it can be seen that GO/CNT/SBL aerogel has good compression resistance.

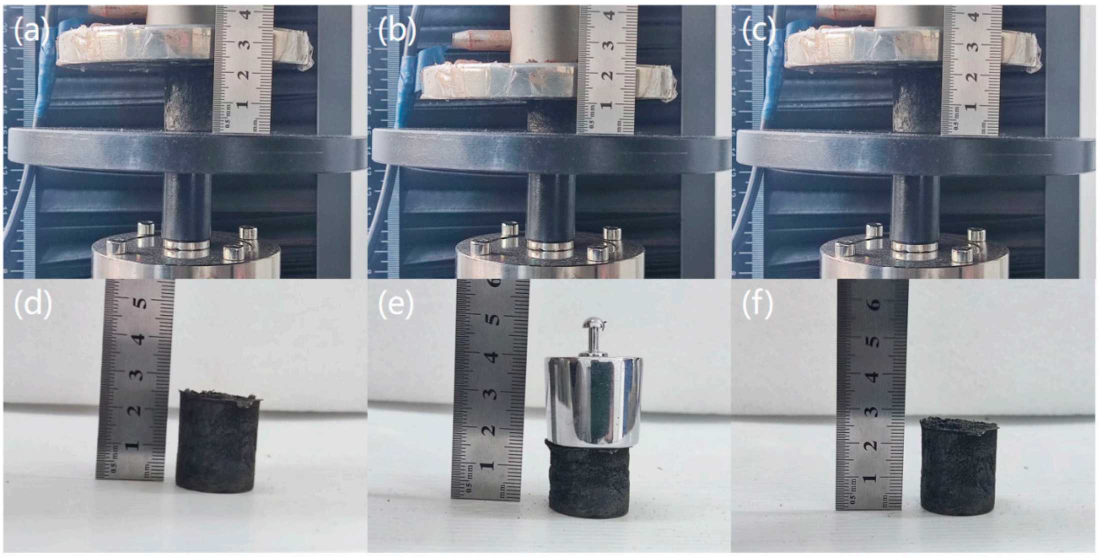

Figure S1. Compression and recovery process of GO/CNT/SBL aerogel.

Figure S2 shows the compression curves of GO/CNT/SBL aerogel prepared by different curing methods. Among these samples, the aerogel without vulcanizing agent and the aerogel without pre-vulcanization completely collapsed during the fifth compression, and the partial collapse of the aerogel without promoting agent could not be restored to its original height, while the aerogel prepared normally did not collapse and almost recovered to its original height. As can be seen from the figure, although the aerogel without vulcanization can withstand a large maximum stress, the stress loss is also the most obvious, while the aerogel with normal pre-vulcanization and vulcanization not only has a large maximum stress, but also a relatively small stress loss. Therefore, it can be concluded that the GO/CNT/SBL aerogel after pre-vulcanization and vulcanization not only has high strength, but also has excellent

compressive resistance.

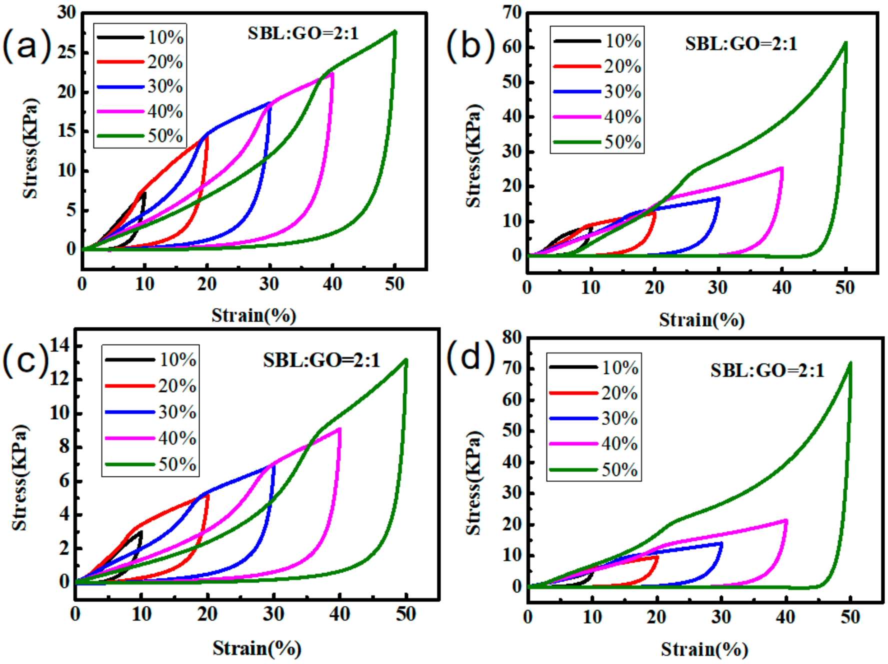

Figure S2. Compression curves of GO/CNT/SBL aerogel (a) Prevulcanization and vulcanization, (b) No Prevulcanization or vulcanization, (c) No accelerant added but prevulcanization and vulcanization, (d) No Prevulcanization but vulcanization).

Figure S3 shows the DSC curves of GO/CNT/SBL aerogel prepared with different ratios and and GO/CNT/SBL aerogel grafted with PEI. It can be seen from the figure that the glass transition temperature of GO/CNT/SBL aerogel prepared with different ratios is around 60°C, without significant change, indicating that the addition of GO and CNT does not affect the glass transition temperature of the styrene-butadiene latex. Compared with the aerogel prepared with ungrafted PEI raw materials, the glass transition temperature of the GO/CNT/SBL aerogel grafted with PEI changed from 62°C to 84°C, indicating that dthe higher molecular weight of PEI resulted in crystallization during the heating process, which increased the glass transition temperature of the graft PEI GO/CNT/SBL aerogel.

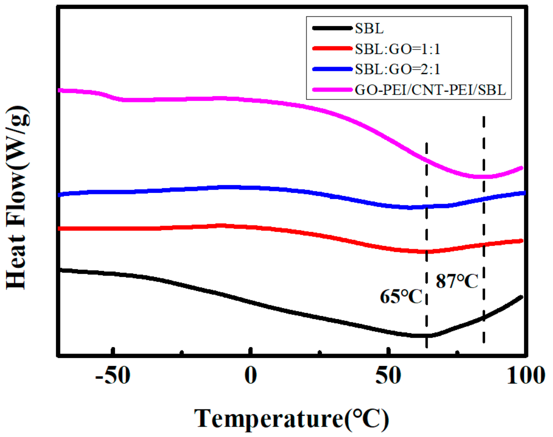

Figure S3. DSC curves of various samples: SBL, GO/CNT/SBL aerogel, GO-PEI/CNT-PEI/SBL aerogel.

Based on calculations, it can be observed from Figure S4 that the BET surface area of the GO/CNT/SBL aerogel and GO-PEI/CNT-PEI/SBL aerogel are 38.96 m2/g and 39.97 m2/g, respectively, while the pore volume is 0.428 cm3/g and 0.439 cm3/g, respectively, with no significant change between them. Therefore, the BET curves demonstrate that the graft of PEI did not modify the surface area of GO and the pore size of the aerogel.

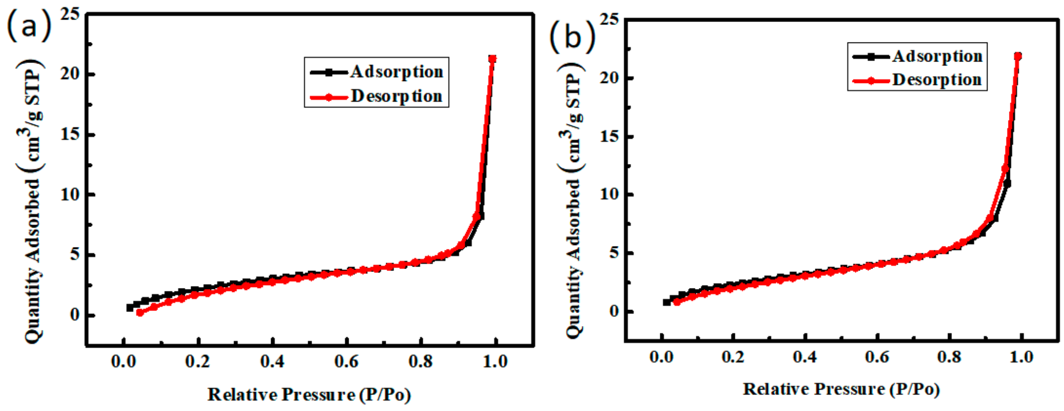

Figure S4. BET curves of various samples: (a) GO/CNT/SBL aerogel, (b) GO-PEI/CNT-PEI/SBL aerogel.
